# ACL-reconstructed and ACL-deficient individuals show differentiated trunk, hip, and knee kinematics during vertical hops more than 20 years post-injury

**DOI:** 10.1007/s00167-017-4528-4

**Published:** 2017-03-23

**Authors:** Jonas L. Markström, Eva Tengman, Charlotte K. Häger

**Affiliations:** Department of Community Medicine and Rehabilitation, Physiotherapy, Umeå University, 901 87 Umeå, Sweden

**Keywords:** Anterior cruciate ligament, Treatment, Long-term, Movement strategy, One-leg vertical hop

## Abstract

**Purpose:**

Little is known regarding movement strategies in the long term following injury of the anterior cruciate ligament (ACL), and even less about comparisons of reconstructed and deficient knees in relation to healthy controls. The present purpose was to compare trunk, hip, and knee kinematics during a one-leg vertical hop (VH) ~20 years post-ACL injury between persons treated with surgery and physiotherapy (ACL_R_), solely physiotherapy (ACL_PT_), and controls (CTRL). Between-leg kinematic differences within groups were also investigated.

**Methods:**

Sixty-six persons who suffered unilateral ACL injury on average 23 ± 2 years ago (32 ACL_R_, 34 ACL_PT_) and 33 controls performed the VH. Peak trunk, hip, and knee angles during Take-off and Landing phases recorded with a 3D motion capture system were analysed with multivariate statistics.

**Results:**

Significant group effects during both Take-off and Landing were found, with ACL_PT_ differing from CTRL in Take-off with a combination of less knee flexion and knee internal rotation, and from both ACL_R_ and CTRL in Landing with less hip and knee flexion, knee internal rotation, and greater hip adduction. ACL_R_ also presented different kinematics to ACL_PT_ and CTRL in Take-off with a combination of greater trunk flexion, hip flexion, hip internal rotation, and less knee abduction, and in Landing with greater trunk flexion and hip internal rotation. Further, different kinematics and hop height were found between legs within groups in both Take-off and Landing for both ACL groups, but not for CTRL.

**Conclusion:**

Different kinematics for the injured leg for both ACL groups compared to CTRL and between treatment groups, as well as between legs within treatment groups, indicate long-term consequences of injury. Compensatory mechanisms for knee protection seem to prevail over time irrespective of initial treatment, possibly increasing the risk of re-injury and triggering the development of osteoarthritis. Detailed investigation of movement strategies during the VH provides important information and a more comprehensive evaluation of knee function than merely hop height. More attention should also be given to the trunk and hip in clinics when evaluating movement strategies after ACL injury.

**Level of evidence:**

Prospective cohort study, Level II.

## Introduction

Anterior cruciate ligament (ACL) injuries are very common in sports and occur mainly in non-contact situations with multidirection knee loading in eccentric movements [12]. Besides its primary function of restraining anterior tibial translation, the ACL simultaneously prevents excessive rotation of the tibia relative to the femur [2]. Kinematic alterations have been reported in the short term post-ACL injury during demanding hop activities [7, 19], but have been less investigated in the longer term and with inconclusive results [27, 30]. Consequences of such kinematic alterations may include mechanical instability, further triggering the development of osteoarthritis (OA) due to altered loadings in the knee joint and thus may affect quality of life [2].

Human movements are characterized by numerous degrees of freedom for coordination and control with multiple rotations and translations from several joints occurring simultaneously. In kinematic analyses, some of these variables will be related to each other, thus providing a set of *n* variables that may reflect underlying dimensions [5]. These dimensions may be detected using multivariate analyses, thus providing a more accurate description of consequences after ACL injury than if using univariate methods. Such analyses are warranted also in the long term post-ACL injury during demanding knee tasks related to everyday life in order to fully understand the impact of an injury. A suitable task is the vertical hop (VH), which is commonly used in clinical environments and considered an important dynamic movement in physical activity [23]. The VH has also been evaluated in research and showed the highest overall sensitivity and accuracy of five frequently used hop tests, with very good to excellent reliability for hop height [11]. To our knowledge, there is no study examining reliability of kinematics specifically for the VH. Excellent to good reliability for kinematics has, however, been shown for the similar drop-vertical jump [15] and single-leg landing tasks [1]. Vandenberg et al. [28] recently pointed out the importance of considering kinematics of the hip, rather than only the knee, for ACL-injured persons. Despite a high hop height and acceptable limb symmetry index (LSI) for the knee, there might be a changed combined movement strategy that may be possible to detect and characterize by using kinematics and applying multivariate statistics.

The main aim of the present study was therefore to for the first time present an investigation of combined kinematics for the trunk, hip, and knee in the long term (>20 years on average) post-unilateral ACL injury for persons treated with either surgery and physiotherapy (ACL_R_) or with solely physiotherapy (ACL_PT_), and compared to a control group (CTRL), when performing the VH. A second aim was to investigate kinematics between legs within groups. Based on previously reported results of functional tasks in the same study population [10, 27], it was anticipated that ACL_PT_ would demonstrate more deviating kinematics compared to CTRL than ACL_R_ to compensate for knee instability, mainly by a reduced active range of motion. It would also seem logical that there would be differences for combined movement strategies between the injured and non-injured legs within both treatment groups. Consideration of combined movement strategies is getting increased clinical awareness, but how to estimate this needs further attention.

## Materials and methods

This cross-sectional research programme involved three groups, consisting of two cohorts from 113 individuals who suffered an ACL injury 17–28 years previously and were treated at two separate hospitals using different treatment approaches, and a control group. A subset of 81 participants with ACL injury was eligible for the present study according to the following inclusion criteria: unilateral ACL injury, not having any surgical total hip or knee replacement (prosthesis), no inflammatory or rheumatic disease or neurological pathology. The ACL-injured persons were treated either with physiotherapy in combination with reconstructive surgery (ACL_R_, *n* = 42) or solely with physiotherapy (ACL_PT_, *n* = 39). Eleven persons declined to participate (9 ACL_R_ and 2 ACL_PT_) due to time constraints and logistical reasons, resulting in 33 ACL_R_ and 37 ACL_PT_ participants. Details of treatments have been presented previously [25]. Briefly, persons in ACL_R_ had physiotherapy treatment for three months before surgery, and all persons had a patellar tendon autograft. A knee brace and crutches were used for 14 weeks after surgery followed by functional exercises with progressively increased demands. For persons in ACL_PT_, a tailored training programme for functional stability was adapted which aimed to achieve an LSI of over 90% for strength and functional tests. Radiological knee OA mostly in stage 1–2, but in some cases up to 4 [14], was detected in ~90% of the participants in both ACL groups at the time of testing. Kinematics of the knee joint were, however, not affected by their OA in a recent publication [27] and were therefore omitted from the analyses of the current study.

One person from ACL_R_ and three persons from ACL_PT_ were excluded from the analyses due to lost marker data in sensitive parts of the VH. A total of 66 ACL-injured persons were therefore included: 32 ACL_R_ (12 women) and 34 ACL_PT_ (13 women). The ACL was injured during team sports (e.g. soccer, floorball) for 53 persons in contact or non-contact, during individual sports (e.g. downhill skiing) for nine persons, and by accident outside of sports for four persons. The CTRL group consisted of 33 persons matched for age and sex (11 women) with no previous knee injuries and normal results from a clinical knee examination. All participants were given written and oral information, and the participants gave their written informed consent according to the Declaration of Helsinki.

### Test protocol

Participants first completed the International Physical Activity Questionnaire (IPAQ), Tegner activity scale, Lysholm questionnaire, Knee Injury and Osteoarthritis Outcome Score (KOOS), and Tampa Scale for Kinesiophobia (TSK), as described elsewhere [25]. Tibial anterior translation was measured with KT1000 arthrometer (Medmetric Corporation, San Diego, CA, USA) at an anterior pull force of 30 lb. Body height and mass were measured to calculate body mass index (BMI). The test procedure began with a 6-min warm-up on a bicycle ergometer at moderate intensity. The VH was performed barefoot on a custom-built force plate with the participants initially standing upright on one leg with arms held across their chest before hopping vertically upwards as high as possible and landing with the same leg on the force plate while maintaining balance. A successful trial required the participant to stand stable after landing for about 2 s without putting the contralateral foot down or removing their arms from their chest. Participants had one to three practice hops and then performed three to four trials on each leg. ACL-injured persons started on the non-injured leg, and CTRL started on the dominant leg and then alternate between legs. Quadriceps and hamstrings strength was then assessed but presented in an earlier paper [26]. The dominant leg was defined as the preferred leg for kicking a ball. The same physiotherapist (ET) instructed all participants.

### Data collection

Kinematics were captured at 240 Hz using a motion capture system with eight cameras (Oqus^®^, Qualisys AB, Gothenburg, Sweden) with markers placed bilaterally on the acromion, clavicle, iliac crest, anterior superior iliac spine, greater trochanters, lateral/medial femoral epicondyles, patellas, tuberositas tibia, fibula head, lateral/medial malleoli, lateral/medial foot, on the sternum, and between the posterior superior iliac spines. Three-marker rigid clusters on thighs and shanks were used in order to reduce soft tissue artefacts and increase reliability and precision [9]. Events used for analyses were set using the custom-made force plate (Department of Biomedical Engineering and Informatics, Umeå University Hospital, Sweden) registering ground reactions at 1200 Hz synchronized with the motion capture system.

### Data analysis

The highest VH for each participant was used in the statistical analyses. The injured (I) leg of persons in the ACL groups was compared to the non-dominant (ND) leg of CTRL, henceforth referred to as I/ND leg, and the non-injured (NI) leg was compared to the dominant (D) leg, henceforth referred to as NI/D leg. Two phases were investigated: (1) *Take*-*off*, defined from peak knee flexion to force signal registration <10 N, and (2) *Landing*, defined from force signal registration >10 N to peak knee flexion. The outcome variables were peak angles of trunk flexion, hip flexion, hip adduction, hip internal rotation, knee flexion, knee abduction, and knee internal rotation. Trunk flexion was defined as movement of the trunk segment relative to the vertical axis in the global coordinate system, and hip and knee joint angles were defined as movement of the distal segment relative to the proximal. The software Qualisys Track Manager (Qualisys AB, Gothenburg, Sweden, version 2.2) and Visual3D (v.5.02.19, C-Motion Inc. Germantown, MD, USA) were used for data processing. An eight-segment rigid body model consisting of feet, shanks, thighs, pelvis, and trunk was constructed, with joint centre calculations based on a six-degrees-of-freedom model [9]. Hop height was calculated using the centre of mass from this model between normal standing and at the highest point of the VH. Data were filtered at 15 Hz with a critically damped digital filter before further calculations. The project was approved by the Regional Ethical Review Board in Umeå (Dnr. 08-211 M).

### Statistical analyses

Hop performances within and between groups were investigated with paired *t* tests and one-way ANOVAs with Bonferroni post hoc tests if significant. Multivariate ANOVAs (MANOVAs) were used to investigate kinematics between groups, and repeated measures MANOVAs were used within subjects. Since BMI differed between treatment groups and CTRL, correlations between BMI and kinematic variables in the sagittal plane were investigated (Pearson), but presented no significant correlations. The effect of BMI as a covariate in MANCOVA was also investigated for the same variables, although with no differences in results. BMI was therefore omitted as a covariate. Significant MANOVAs were investigated with direct discriminant analyses (DISCRIM) [5], with correlations >0.32 interpreted. Within-subject contrasts were used for between-leg comparisons. Assumptions of absence of multivariate outliers and multicollinearity, linearity, and homogeneity of variance–covariance were met. A power analysis based on pilot tests including five ACL-injured persons and five controls suggested that 32 persons/group were needed for a power of 80% to detect a significant difference in knee joint flexion angle between groups with a variance of 10° and a significance level of 5% (which was also used in analyses). All statistical analyses were conducted using IBM SPSS (version 22, Armonk, New York, USA).

## Results

**Fig. 1:**
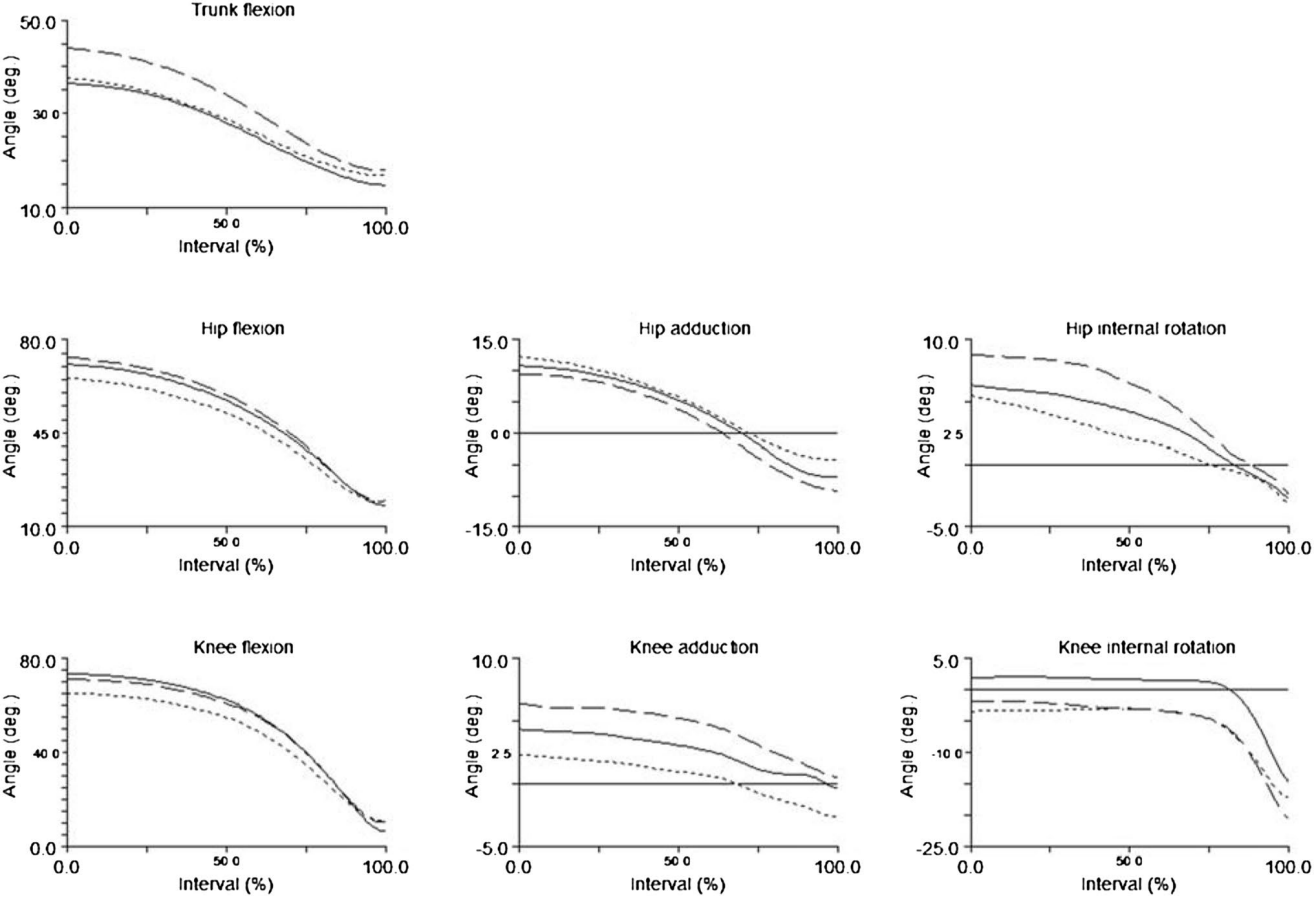
Ensemble mean trunk, hip, and knee kinematics during the time-normalized Take-off phase of the injured leg for ACL groups, and of the non-dominant leg for CTRL. Flexion, adduction, and internal rotation in positive angles. Angle at 0° is marked with a *horizontal line*. Each interval on the *Y-*axis is equal to 5°. *Dashed line* for ACL_R_, *dotted line* for ACL_PT_, *solid line* for CTRL

**Fig. 2:**
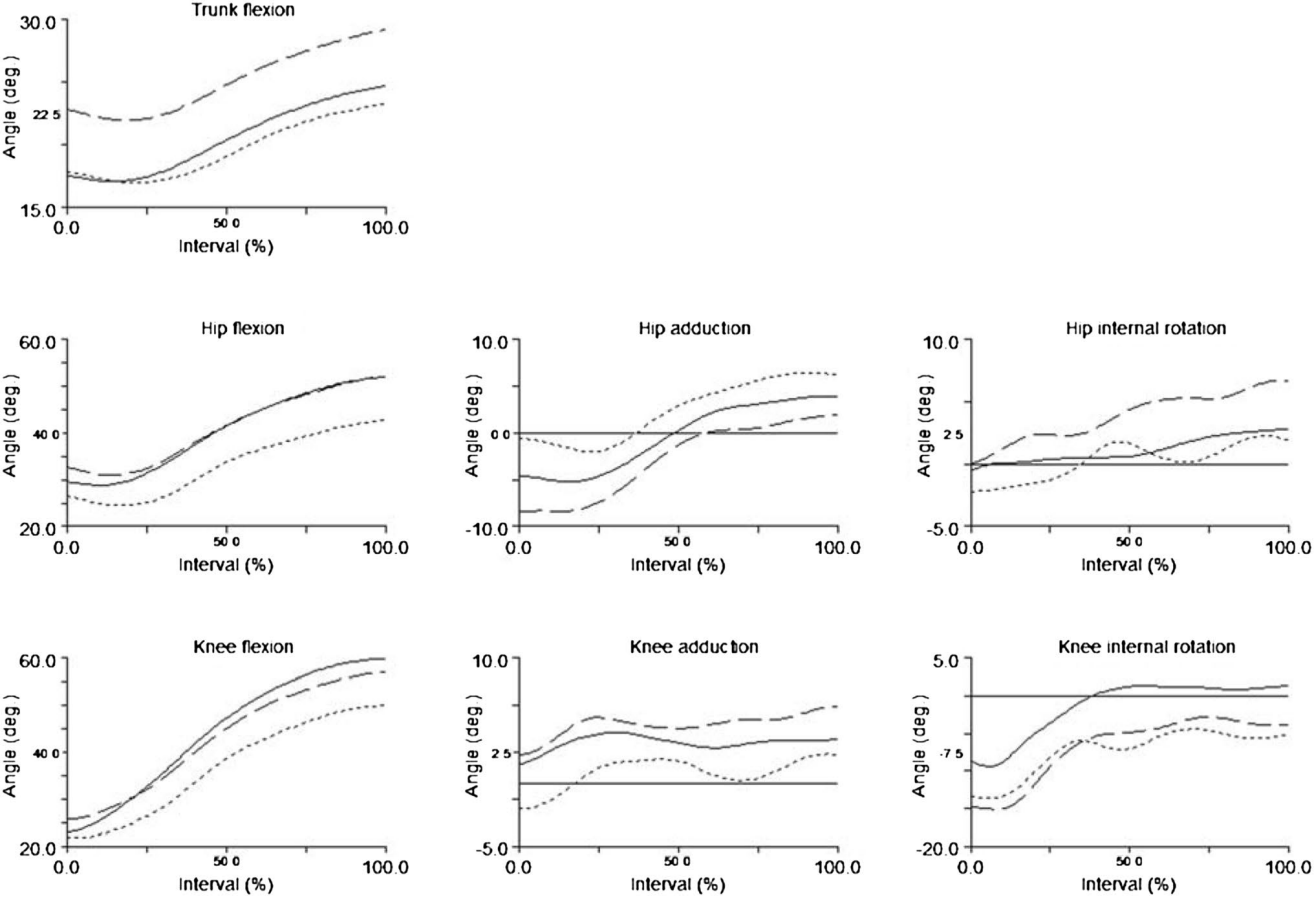
Ensemble mean trunk, hip, and knee kinematics during the time-normalized Landing phase of the injured leg for ACL groups, and of the non-dominant leg for CTRL. Flexion, adduction, and internal rotation in positive angles. Angle at 0° is marked with a *horizontal line*. Each interval on the *Y*-axis is equal to 5°. *Dashed line* for ACL_R_, *dotted line* for ACL_PT_, *solid line* for CTRL

Background data and hop height are presented in Table 1. Both treatment groups had significantly greater laxity for the affected compared to the non-affected leg (*p* < 0.001 for both groups), with ACL_PT_ showing a significantly greater difference compared to ACL_R_ (95% CI of 1.6–4.3 mm difference, *p* < 0.001). A significant effect of group on hop height for I/ND comparisons was found (*p* = 0.007), with ACL_PT_ having a lower hop height than both ACL_R_ (95% CI of 0.001–0.05 m difference, *p* = 0.04) and CTRL (95% CI of 0.01–0.05 m difference, *p* = 0.01), while no differences were shown between ACL_R_ and CTRL. Both treatment groups had lower hop height with their injured compared to their non-injured leg (*p* = 0.006 for ACL_R_ and *p* = 0.001 for ACL_PT_). Average kinematic angles are presented in Figs. 1 and 2 for Take-Off and Landing phases, respectively. Peak angles for trunk, hip, and knee kinematics in Take-off and Landing for I/ND and NI/D legs are presented in Table 2.

**Table 1:** Background data and hop height for each group

	ACL_R_ *N* = 32	ACL_PT_ *N* = 34	CTRL *N* = 33
BMI	27.1 (3.3)^a^	28.7 (4.3)^a^	24.6 (2.5)
Age (years)	45.5 (4.6)	47.6 (5.9)	46.7 (5.0)
Years since ACL injury	23.8 (2.7)	23.1 (1.3)	–
Years since ACL surgery	20.1 (1.5)	–	–
Anterior translation I/ND–NI/D leg (mm)	2.0 (2.7)^a,b^	4.9 (2.9)^a^	−0.1 (1.1)
Hop height I/ND leg (m)	0.20 (0.04)^b,c^	0.17 (0.03)^a,c^	0.20 (0.04)
Hop height NI/D leg (m)	0.21 (0.04)	0.19 (0.04)	0.20 (0.03)
LSI hop height (%)	94 (13)	91 (14)^a^	104 (23)
KOOS_SYMPTOMS_ (score)	84 (100)^a^	75 (61)^a^	100 (7)
KOOS_PAIN_ (score)	82 (58)^a^	89 (50)^a^	100 (6)
KOOS_ADL_ (score)	89 (58)^a,b^	98 (62)^a^	100 (0)
KOOS_SPORT_ (score)	50 (100)^a,b^	75 (100)^a^	100 (10)
KOOS_QOL_ (score)	49 (22)^a,b^	69 (94)^a^	100 (13)
TSK (score)	33 (55)	32 (27)	–
IPAQ (score)	2391 (1591)	2254 (2045)	2570 (2097)
IPAQ (assigned score)	2 (2)	2 (2)	2 (2)
Tegner pre-injury (score)	9 (7)^b^	9 (6)	–
Tegner 20 years (score)	4 (4)^a^	4 (5)^a^	6 (4)
Lysholm 20 years (score)	81 (64)^a^	73 (61)^a^	100 (0)

Data presented in mean (SD) with the exception of questionnaires (analysed with nonparametric statistics) with scores presented in median (range). LSI, limb symmetry index being the ratio between I/NI leg for ACL groups and ND/D leg for CTRL; I/ND leg, injured leg for ACL groups and non-dominant leg for CTRL; NI/D leg, non-injured leg for ACL groups and dominant leg for CTRL. For more details of questionnaires and hop performances, see Tengman et al. [25]

^a^Significant difference observed to CTRL

^b^Significant difference observed to ACL_PT_

^c^Significant difference observed to the NI leg within groups

**Table 2:** Peak angles of trunk, hip, and knee joints presented in mean (SD)

	I/ND leg		NI/D leg	
	TO	LA	TO	LA
Trunk forward flexion (°)				
ACL_R_	44.2 (12.7)^a^	30.0 (8.9)^a^	40.6 (11.2)	26.0 (9.7)
ACL_PT_	37.7 (11.0)^a^	23.8 (7.7)	35.2 (11.5)	23.0 (9.4)
CTRL	39.1 (9.6)	23.6 (9.9)	36.6 (9.4)	24.8 (9.7)
Hip flexion (°)				
ACL_R_	73.3 (15.4)	52.44 (12.0)	71.7 (12.4)	50.1 (11.9)
ACL_PT_	65.4 (16.7)	43.0 (11.5)	63.1 (15.5)	44.8 (13.1)
CTRL	70.7 (12.9)	52.0 (10.7)	72.3 (12.5)	49.8 (10.9)
Hip adduction (°)				
ACL_R_	9.8 (6.6)	3.3 (7.8)	10.6 (6.8)	4.7 (6.9)
ACL_PT_	12.3 (7.5)	7.8 (7.6)	10.2 (6.9)	5.8 (8.7)
CTRL	11.0 (5.6)	5.5 (5.6)	11.0 (6.9)	5.6 (8.0)
Hip internal rotation (°)				
ACL_R_	10.6 (8.2)^a^	10.6 (8.1)	6.7 (6.4)	7.6 (7.0)
ACL_PT_	6.6 (9.3)^a^	6.5 (7.8)	2.9 (8.4)	4.0 (8.5)
CTRL	7.6 (6.3)	7.5 (7.5)	6.1 (7.1)	6.8 (9.1)
Knee flexion (°)				
ACL_R_	71.3 (10.5)^a^	57.0 (10.6)	74.3 (8.7)	58.1 (9.2)
ACL_PT_	65.3 (9.7)	50.0 (9.3)^a^	66.6 (7.9)	54.7 (8.9)
CTRL	73.6 (8.8)	59.9 (9.1)	74.1 (8.1)	58.5 (9.0)
Knee abduction (°)				
ACL_R_	1.2 (5.1)	0.0 (4.7)	2.1 (5.1)	1.7 (5.1)
ACL_PT_	4.1 (5.5)	3.4 (6.3)	5.7 (4.1)	4.8 (5.2)
CTRL	2.3 (5.0)	1.6 (6.2)	2.0 (4.9)	0.8 (5.1)
Knee internal rotation (°)				
ACL_R_	−0.1 (7.0)	0.4 (6.8)	1.4 (6.8)	2.8 (6.7)
ACL_PT_	−1.5 (5.5)^a^	−1.6 (6.7)^a^	2.0 (7.0)	3.3 (7.8)
CTRL	4.4 (7.3)	5.1 (7.0)	1.3 (5.0)	2.8 (5.3)

Negative values in knee internal rotation denote external rotation. I/ND leg, injured leg for ACL groups and non-dominant leg for CTRL; NI/D leg, non-injured leg for ACL groups and dominant leg for CTRL; TO, Take-off phase; LA, Landing phase

^a^Significant within-subject difference observed to NI leg

### Kinematics for injured/non-dominant leg in Take-off and Landing phases

**Fig. 3:**
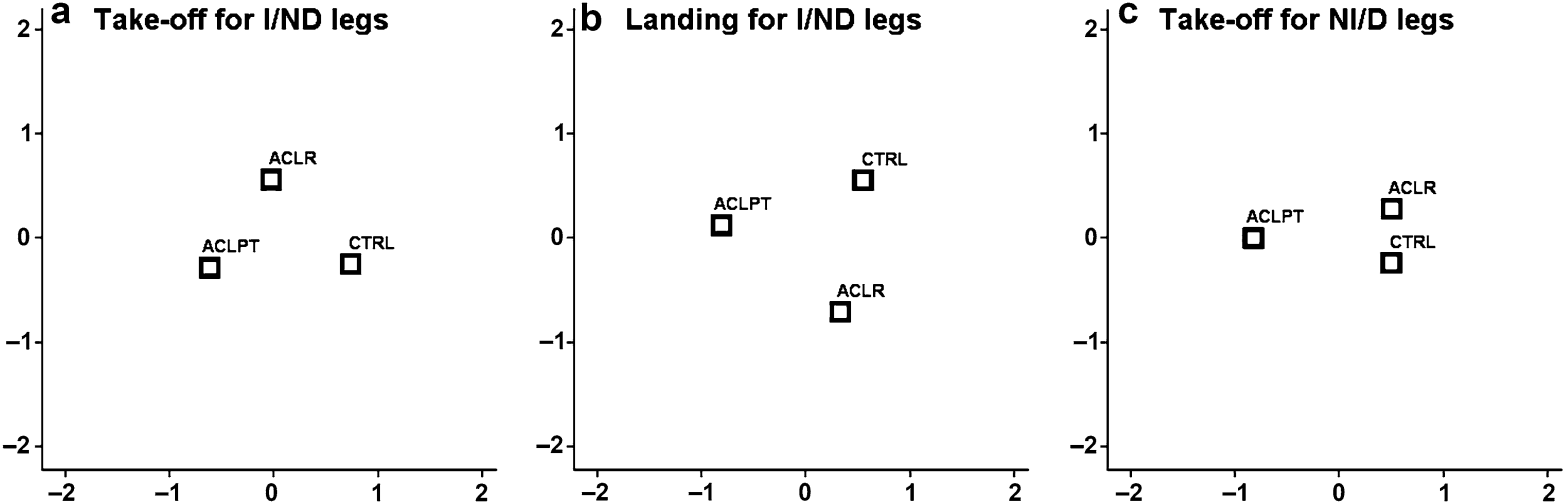
Kinematic representation of discriminant function plots for all groups. Distances of group centroids on the *x*-axis and *y-*axis are of interest in **a** and **b**, and the distance on the *x-*axis is of interest in **c**. Combinations of differences in kinematic variables between the groups correlate with these functions, thus separating the group centroids. These are in descending order with the highest correlation first: in **a** knee internal rotation and knee flexion contributes to distances in *x*-axis, and trunk flexion, hip internal rotation, knee abduction, and hip flexion to distances in *y*-axis; in **b** knee flexion, hip flexion, knee internal rotation, and hip adduction contribute to distances in *x*-axis, and trunk flexion and hip internal rotation contribute to distances in *y*-axis; in **c** knee flexion, knee abduction, hip flexion, and hip internal rotation contribute to distances in *x-*axis

Take-off: significant kinematic differences were found for I/ND legs between groups during Take-off (*p* < 0.001). Follow-up analysis with DISCRIM revealed two significant discriminant functions (*p* < 0.001 with canonical *R* ^2^ = 0.26 and *p* = 0.018 with canonical *R* ^2^ = 0.15). High correlations were found for knee internal rotation and knee flexion to the first function (*r* = 0.63 and 0.59, respectively) and for trunk flexion, hip internal rotation, knee abduction, and hip flexion for the second function (*r* = 0.59, 0.49, 0.43, and 0.40, respectively). The discriminant function plot in Fig. 3a shows the first function separating ACL_PT_ from CTRL, and the second function separating ACL_R_ from ACL_PT_ and CTRL. That is, ACL_PT_ demonstrated a combination of less internal rotation of the knee (i.e. negative angles in Table 2 denote greater external rotation) and less knee flexion than CTRL, and ACL_R_ demonstrated a combination of greater trunk flexion, greater hip internal rotation, less knee abduction, and greater hip flexion than both ACL_PT_ and CTRL.

Landing: significant differences were found for I/ND legs between groups during Landing (*p* < 0.001). DISCRIM revealed two significant discriminant functions (*p* < 0.001 with canonical *R* ^2^ = 0.28 and *p* = 0.001 with canonical *R* ^2^ = 0.22). High correlations were found for knee flexion, hip flexion, knee internal rotation, and hip adduction to the first function (*r* = 0.71, 0.62, 0.57, and −0.33, respectively) and for trunk flexion and hip internal rotation for the second function (*r* = −0.60 and −0.35, respectively). The discriminant function plot in Fig. 3b shows the first function separating ACL_PT_ from both ACL_R_ and CTRL and the second function separating ACL_R_ from both ACL_PT_ and CTRL. Thus, ACL_PT_ demonstrated a combination of less knee and hip flexion, less knee internal rotation (i.e. greater external rotation), and greater hip adduction than ACL_R_ and CTRL, and ACL_R_ demonstrated a combination of greater trunk flexion and hip internal rotation than both ACL_PT_ and CTRL.

### Kinematics in non-injured/dominant leg in Take-off and Landing phases

Significant differences were also found for NI/D legs between groups during Take-off (*p* = 0.004), but not in Landing (n.s.). DISCRIM revealed one significant discriminant function (*p* = 0.002 with canonical *R* ^2^ = 0.27) with high correlations with knee flexion, knee abduction, hip flexion, and hip internal rotation (*r* = 0.72, 0.62, 0.52, and 0.37, respectively). The discriminant function plot in Fig. 3c shows ACL_PT_ differing from both ACL_R_ and CTRL by demonstrating less knee flexion, greater knee abduction, less hip flexion, and less hip internal rotation.

### Leg comparisons within groups

Between-leg comparisons showed significant differences for ACL_R_ and ACL_PT_ in both Take-off (*p* < 0.001 and *p* = 0.004, respectively) and Landing (*p* = 0.026 and 0.020, respectively), but not for CTRL in any of the phases (n.s.). See Table 2 for within-subject contrasts.

## Discussion

 The main finding of the present study is that the combined movement strategies of the trunk, hip, and knee differed for the two treatment groups and compared to healthy controls during the VH. More specifically, ACL_PT_ demonstrated kinematics characterized by reduced hip flexion, knee flexion, and knee rotation, while ACL_R_ presented the greatest trunk flexion, hip flexion, and hip rotation. The greater difference between legs in anterior tibial laxity for ACL_PT_ may explain these movement strategies. ACL_PT_ also presented lower hop height than ACL_R_ and CTRL. Despite this, there were similar scores for the two ACL groups on Tegner activity scale and IPAQ, and higher scores on the KOOS subscales ADL, SPORT, and QOL for ACL_PT_ compared to ACL_R_, indicating that ACL_PT_ had not adapted a more restricted movement strategy compared to ACL_R_. This may indicate that knee scores alone do not capture the entire picture with regard to knee function evaluation, and thus, motion analysis provides additional value.

In the absence of an ACL, knee stability relies on the remaining static and dynamic stabilizers to restrict the tibia from excessive anterior displacement and rotation. Since the ACL has an oblique medial orientation from the femur to the tibia [2], a ruptured ACL may result in a more internally rotated tibial position during passive motion. Indeed, in knees where the ACL has been removed, an increased internal rotation has been shown during application of an axial tibial force [16]. In the present study, the observed less pronounced knee internal rotation, in fact an externally rotated knee, in combination with less knee flexion in Take-off for ACL_PT_ compared to CTRL, may therefore be a knee-protective strategy to avoid positions of internal rotation where give way might occur. The same strategy was found during Landing, although with the addition of less hip flexion and greater hip adduction when compared to both ACL_R_ and CTRL. Since an internal tibial torque also induces a coupled anterior tibial translation relative to the femur [13], this further supports a protective avoidance strategy of potentially risky knee positions for ACL_PT_. Similar results of reduced knee sagittal plane movement for ACL-deficient persons compared to ACL_R_ and CTRL groups have been shown in landings of a one-leg hop for distance [22]. This movement strategy seems not to be explained by quadriceps strength since no differences were shown between any of these groups for knee extension strength, although both treatment groups had lower knee flexor strength than controls. In contrast, in our study population only the concentric knee extension strength, but not concentric flexion strength, was lower for both ACL_PT_ and ACL_R_ compared to CTRL, as presented previously [26].

ACL_R_ adapted a strategy where they increased trunk and hip flexion compared to both ACL_PT_ and CTRL. Increased trunk flexion was indeed observed in both phases for the injured compared to the non-injured leg for ACL_R_. Similar results with greater trunk flexion between legs for ACL-reconstructed persons in another study further revealed a forward shift of the centre of pressure, which resulted in a more anterior position of the ground reaction force vector in relation to the hip, knee, and ankle joint axes [18]. This strategy shifted the joint moment from the knee to the adjacent joints. Instructions to land with increased trunk flexion also result in increased hip and knee flexion, but do not influence movement in the frontal or transversal planes, when compared to a preferred landing strategy during the vertical drop landing [4]. On the other hand, a landing strategy with a more upright trunk flexion results in increases in peak vertical ground reaction force, peak knee extensor moment, hip moment, and quadriceps amplitude [24]. A small knee flexion angle also increases the patellar tendon insertion angle and decreases the hamstrings insertion angle, which results in an increased anterior tibial shear force for a given anterior tibial shear load [4]. The ACL_PT_ group would therefore seem to have adopted a movement strategy that restricts movement, although provokes an anterior tibial translation when compared to the other groups. This movement strategy was particularly evident when comparing their injured leg to the non-injured leg in Landing, demonstrating less knee flexion and knee internal rotation for the injured leg. In addition, the altered kinematics also for the non-injured leg in ACL_PT_ compared to both ACL_R_ and CTRL further indicate a crossover effect displayed to a greater extent than for ACL_R_. The restrained movement strategy for ACL_PT_ for both legs may indicate a greater vulnerability to challenging loadings of the knee in one-leg hops [21], possibly also increasing the risk of injury for the intact ACL of the other leg, and contributing to development of OA [2]. Of particular relevance here may be the average differences in knee rotation of 5.9° in Take-off and 6.7° in Landing which were shown for ACL_PT_ compared to CTRL for I/ND legs. These are above the value of 5° shown to accelerate cartilage thinning [3], which may be detrimental to future knee health. Particular consideration should be given to the coupled average differences in knee flexion of 8.3° in Take-off and 9.9° in Landing between the same groups. For comparison, there were non-significant average values of knee flexion and knee rotation for ACL_R_ and CTRL in Take-off of 2.3° and 4.5°, respectively, and in Landing of 2.9° and 4.7°, respectively. Hop tests with a greater emphasis on rotational demands may enhance this difference for ACL_R_ compared to CTRL due to rotational instability that seems to remain after ACL reconstruction [10, 29]. Previously reported results from the same population show that both treatment groups have different kinematics compared to the same controls also in the two-leg squat and side hop tests with knee rotational instability [10] and in the one-leg hop for distance where less knee flexion and knee internal rotation were shown [27]. The results of the present study further corroborate these earlier findings but now extend the analysis of movement strategies to also include trunk and hip movements using multivariate methods.

There are very few long-term kinematic studies, and the existing ones do not indicate any differences between ACL-injured persons and controls. von Porat et al. [30] reported similar knee kinematic and kinetic results for ACL-injured persons and matched healthy controls during gait, step up, and crossover hop tests at 16 years post-ACL injury. Ortiz et al. [20] also reported similar hip and knee kinematic outcomes between physically active women with ACL reconstruction compared to healthy, non-injured women in drop jump and up-down tasks. However, only 12 persons (six treated with surgery, six without) participated in the 16-year follow-up and just 13 ACL-injured persons, with a mean time of 7.2 years (range of 1–16 years) after reconstruction, participated in the latter study. These smaller studies with mixed groups might be under-powered, and thus, the inconsistent results may depend on study populations, group sizes, and variations in assessment and follow-up times.

A general insight into clinical importance is that knee scores alone most likely do not capture the entire picture with regard to evaluation of knee function. Analysis of movement strategies certainly provides additional value, especially when evaluating treatment. A VH test is easy to administer in the clinic, is not time-consuming, enables comparisons of biomechanical asymmetries between injured and non-uninjured sides, and can be used to determine progress in rehabilitation. Different kinematics for both ACL groups are of clinical relevance since instability may exist irrespective of treatment. Both ACL_PT_ and ACL_R_ had >90% LSI for hop height, despite demonstrating different movement patterns and low self-estimated knee function. Interpretation of results based on LSI scores or absolute measures from hop performances should therefore be analysed with caution when evaluating knee function if used without consideration of other knee function measures. Similar results have been found for ACL-injured persons achieving >90% in dynamic tests despite presenting altered kinematics [19] or having self-estimated unstable knees [6, 30]. Hopefully, better and simpler motion analysis of movement strategies in the clinics may be developed based on more detailed analyses such as in the present study. Our results highlight the importance of incorporating the trunk and hip when evaluating movement strategies for ACL-injured persons in the clinics rather than only focusing on the knee joint.

Some limitations of the present study should be acknowledged. The use of skin markers implies risks of soft tissue artefacts influencing validity and reliability. To reduce this, clusters of markers were used on areas with large muscle groups for better approximations. Soft tissue artefacts seem to be similar between subjects and hence should not mask group differences [8]. Comparisons between the different treatment groups could also be questioned as this was not an RCT. Long-term kinematic studies are, however, very rare, and the biomechanical conditions from these cohorts were described without necessarily drawing direct conclusions about treatment recommendations. It should be mentioned that major improvements in AC reconstruction techniques has occurred during the past 20 years and need to be considered in relation to our results. Multiple health-related aspects over the years may also play a role, such as the precise impact and course of OA that may vary [17]. However, no effects of the degree of radiological knee OA on knee kinematics was found in an earlier report on the same study population [25]. The higher Tegner activity score for CTRL compared to both treatment groups could be questioned, but was partly explained by the high score assigned to occasional participation in downhill skiing during leisure time for many participants as they live in a northern climate. No group differences for IPAQ scores may indicate that the amount of activity does not explain the different movement patterns. Also, since BMI did not present any correlations with or influenced any of the kinematic results, the groups included in this study could be considered comparable for this biomechanical investigation.

## Conclusion

This biomechanical study more than 20 years post-injury of the one-leg vertical hop during Take-off and Landing phases highlights different movement strategies for combinations of trunk, hip, and knee kinematics for the injured side of both ACL-reconstructed and ACL-deficient groups compared to CTRL and between ACL groups. Future controlled studies with stringent clinical and biomechanical assessment involving overall movement strategies of the trunk and leg post-ACL injury are desirable to evaluate type of treatment and course of rehabilitation and would be of relevance for clinical implementation.
